# Neonatal necrotizing enterocolitis: *Clostridium butyricum* and *Clostridium neonatale* fermentation metabolism and enteropathogenicity

**DOI:** 10.1080/19490976.2023.2172666

**Published:** 2023-02-21

**Authors:** Laurent Ferraris, Aurélie Balvay, Deborah Bellet, Johanne Delannoy, Claire Maudet, Thibaut Larcher, Jean-Christophe Rozé, Catherine Philippe, Thierry Meylheuc, Marie-José Butel, Sylvie Rabot, Julio Aires

**Affiliations:** a Université Paris Cité, INSERM, UMR-S 1139, 3PHM, Paris, France; b FHU PREMA « Fighting prematurity, Paris, France; c Université Paris-Saclay, INRAE, AgroParisTech, Micalis Institute, Jouy-en-Josas, France; d INRAE, Oniris, PAnTher, APEX, Nantes, France; e INRAE, UMR 1280, Physiologie des Adaptations Nutritionnelles (PhAN), Université hospitalière de Nantes, Nantes, France

**Keywords:** *Clostridium butyricum*, *Clostridium neonatale*, necrotizing enterocolitis, butyrate, animal model, beta-hydroxybutyryl-CoA dehydrogenase, NEC, NEC-like lesions

## Abstract

Bacterial colonization in the gut plays a pivotal role in neonatal necrotizing enterocolitis (NEC) development, but the relationship between bacteria and NEC remains unclear. In this study, we aimed to elucidate whether bacterial butyrate end-fermentation metabolites participate in the development of NEC lesions and confirm the enteropathogenicity of *Clostridium butyricum* and *Clostridium neonatale* in NEC. First, we produced *C.butyricum* and *C.neonatale* strains impaired in butyrate production by genetically inactivating the *hbd* gene encoding β-hydroxybutyryl-CoA dehydrogenase that produces end-fermentation metabolites. Second, we evaluated the enteropathogenicty of the hbd-knockout strains in a gnotobiotic quail model of NEC. The analyses showed that animals harboring these strains had significantly fewer and less intense intestinal lesions than those harboring the respective wild-type strains. In the absence of specific biological markers of NEC, the data provide original and new mechanistic insights into the disease pathophysiology, a necessary step for developing potential novel therapies.

## Introduction

Necrotizing enterocolitis (NEC) is one of the most severe and life-threatening gastrointestinal diseases in neonatal intensive care units, with often an unpredictable and sudden onset. In high-income countries, NEC incidence has remained constant, ranging from 2% to 7% in infants born at <32 weeks’ gestation and 5% to 22% in those with birth weight <1,000 g.^1,2^ The incidence of NEC is associated with a high mortality rate (20% to 30%); moreover, survivors experience long-term complications.^1,2^ Healthcare management of patients with NEC, both medically and surgically, is associated with significantly higher hospital costs than that of preterm neonates without NEC.^3^ Clinical symptoms and biological markers of NEC are not specific, thus complicating the diagnosis. Moreover, there remain no clear recommendations for NEC prevention.^4^

The susceptibility of preterm neonates to NEC and the disease pathophysiology remain incompletely understood. NEC is a multifactorial disease, with coalescence of three primary factors – the immaturity of the preterm infant, enteral feeding, and intestinal microbiota dysbiosis – that lead to an imbalance between pro-inflammatory and anti-inflammatory mediators.^5,6^ The relative contribution of each factor is unclear, but gut bacterial colonization appears to play a pivotal role in NEC onset.^5,6^ The microbial hypothesis of NEC is supported by the following factors: clustering of cases and outbreaks suggestive of an infective component to etiology; initial clinical management of NEC with antibiotics that participate in improving outcomes; and the fact that NEC cannot be developed in germ-free animals.^7–10^

The alteration of the gut microbiota in infants with NEC compared to healthy infants has been associated with an increased abundance of opportunistic bacteria, mainly Enterobacteriaceae and *Clostridium*.^11–14^ Studies by our and other groups have reported a link between NEC and gut colonization by *Clostridium butyricum, Clostridium neonatale*, or *Clostridium perfringens* in preterm neonates.^14–17^ *Clostridium* spp. have been isolated from the blood, feces, and peritoneal fluids of preterm neonates with NEC.^11–13^ Moreover, their role in NEC pathogenesis has been strengthened in animal models.^11,12^ Indeed in the preterm piglet model, *C. butyricum* and other clostridia species were largely absent in piglets without NEC and significantly overrepresented among colonic mucosal samples from piglets with NEC.^18^ Using germ-free quails fed a lactose diet and monoassociated with *C. butyricum*, we have shown that lactose intestinal fermentation leads to high levels of butyric acid, a prerequisite for the development of cecal NEC-like lesions.^19–22^ Removal of dietary lactose suppresses mucosal damage in animals.^23^ In their study, Smith et al. reported a correlation between the presence of *C. butyricum* and pneumatosis intestinalis in tissue specimens from neonates with NEC.^24^ Pneumatosis intestinalis and portal venous gas are pathognomonic radiographic signs of NEC proposed to be gas production due to bacterial fermentation metabolism.^1^ Altogether, these data suggested that clostridia could lead to gut inflammation and NEC lesions through their metabolic activity.

Studies suggest that excessive and/or abnormal luminal concentrations of short-chain fatty acids (SCFAs), including butyrate, may exceed the physiological capacity of the immature intestinal mucosa of preterm neonates and may damage the intestinal mucosa.^25–30^ Moreover, the overproduction of bacterial metabolites and fermentation of undigested lactose due to functional deficiency of lactase activity in the immature intestine of preterm neonates have been proposed but rarely investigated.^25,26^ Interestingly, the overproduction of bacterial metabolites due to undigested lactose may trigger the inflammatory cascade.^31^ Albeit, butyric acid is essential for normal colonic function, high concentrations have dose-dependent deleterious effects in both cellular and animal models.^19,20,27–30,32^ Elevated fecal butyrate levels have been observed in extreme preterm neonate cases with brain injuries.^33^

The present study aimed to investigate the role of *Clostridium* and fermentation end-product metabolites, particularly butyrate, in NEC pathogenesis. Here, we genetically engineered *C. butyricum* and *C. neonatale* clinical strains isolated from NEC cases to impair their fermentation-end metabolite production and examined their enteropathogenicity in a gnotobiotic quail model of NEC.

## Results

### *C. butyricum* and *C. neonatale* hbd *mutagenesis*

**Figure 1.**
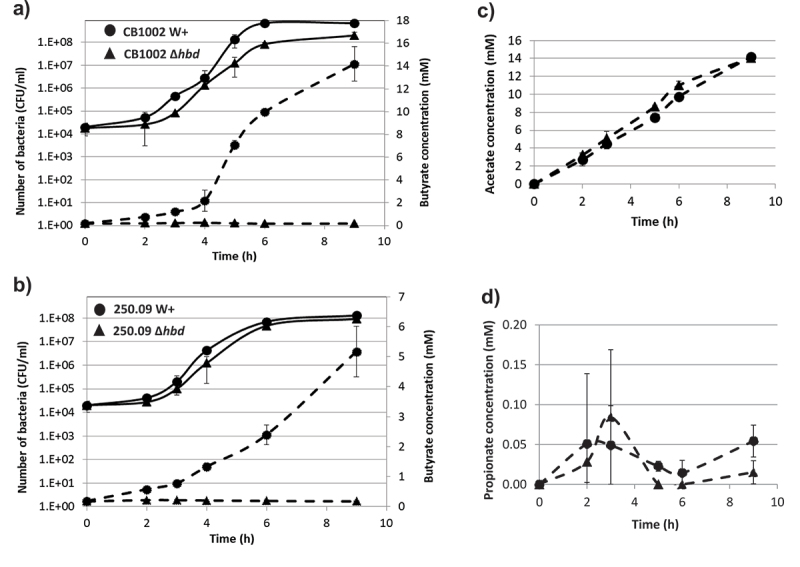
Growth curves (full lines) and butyrate production (dotted lines) of wild-type (●) or *hbd*-knockout (▲) (a) *C. butyricum* CB1002 and (b) *Clostridium neonatale* 250.09 strains. Data are average of three independent experiments.

*C. butyricum* CB1002 and *C. neonatale* 250.09 *hbd*-knockout were obtained using the ClosTron-directed mutagenesis tool.^34^ It allowed the inactivation by homologous recombination of the *hbd* gene encoding a β-hydroxybutyryl-CoA dehydrogenase responsible for the production of end-fermentation metabolites, particularly butyrate (Fig. S1). Insertion of the Group II intron into the *hbd* was verified by PCR and confirmed across the *hbd* and intron–junction using specific primers (Table S1). The *C. butyricum* CbuCB1002-*hbd*::CT and *C. neonatale* Cne250.09-*hbd*::CT knockouts (KO) showed significantly lower butyrate production than their respective wild-type (WT) strains (P = .003 and P = .009, respectively, after 9 h of culture) (Figure 1a,b). The WT and KO strains had similar growth rates (Figure 1a,b). Moreover, for both WT and KO strains, acetate and propionate production rates were similar (P > .05) (Figure 1c,d).

### *C. butyricum* CB 1002 wild-type and hbd-knockout pathogenicity

**Figure 2.**
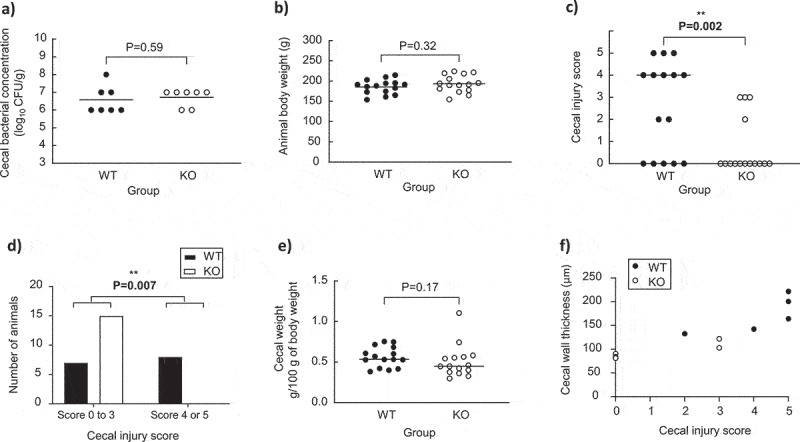
Experimental data of animals’ monoassociated with the wild-type (WT) and knock-out (KO) *C. butyricum* CB1002 strains. (a) Cecal bacterial concentration in quails monoassociated with the WT (n = 7) or KO (n = 7) strains. (b) Body weight of quails monoassociated with the WT (n = 15) or KO (n = 15) strains. (c) Cecal weight of quails monoassociated with the WT (n = 15) or KO (n = 15) strains. (d) Cecal injury score of quails monoassociated with the WT (n = 15) or KO (n = 15) strains. (E) Distribution of the number of quails monoassociated with the WT (n = 15) or KO (n = 15) strains based on the cecal injury score. (f) Correlation between cecal wall thickness and cecal injury score of quails monoassociated with the WT (n = 5) or KO (n = 5) strains. Statistical significance was set at P < .05.

*C. butyricum* CB 1002 WT and KO animal experimental data are presented in Figure 2. In quails monoassociated with the WT and KO strains, the cecal concentrations were not different between both animal groups (Figure 2a). Body weights at the end of the experiment were equivalent between both groups (Figure 2b) and no quail showed any clinical sign of disease. Based on the scoring scale and severity degree of the animal’s cecal lesions (Table S2), the cecal injury score was significantly higher in the WT than in the KO group (Figure 2c). Moreover, the proportion of animals exhibiting at least two NEC-like lesions (scores 4 and 5) was significantly higher in the WT than in the KO group (Figure 2d). The ceca in the WT were not heavier than that in KO group (Figure 2e). Macroscopically, the main lesions in the WT group were cecal hemorrhage foci and wall thickening. Peculiar lesions were observed in two quails of the KO group who had a lesion score of 3 – extensive pneumatosis occurred in the mesentery of these quails; in contrast, the intensity of the mesenteric pneumatosis remained moderate in the five quails of the WT group who displayed this lesion, despite their higher lesion score (4 or 5). Examples of ceca are presented in Fig. S2.

**Figure 3.**
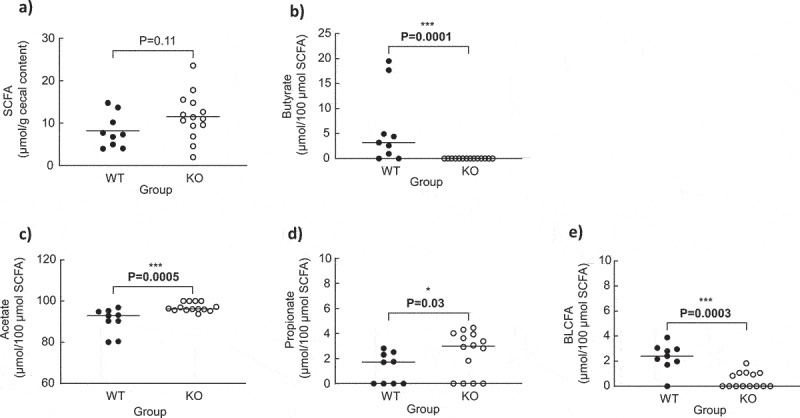
Cecal SCFA, branched long-chain fatty acid (BLCFA), acetate, propionate, and butyrate concentrations in quails monoassociated with the wild type (WT) (n = 9) or knock-out (KO) (n = 14) *C. butyricum* CB1002 strains. Data are individual results with medians. Statistical significance was set at P < .05.

The total cecal SCFA concentrations were similar between the WT and KO groups (Figure 3a). Butyrate was not detected in the cecal content of the quails colonized with the KO strain as opposed to those colonized with WT strain (Figure 3b). Acetate and propionate proportions were higher in the KO than those in the WT groups (Figure 3c,d). In contrast, a higher proportion of branched long-chain fatty acids (BLCFAs) was found in the WT than that in the KO groups (Figure 3e).

### *C. neonatale* 250.09 wild-type and hbd-knockout pathogenicity

**Figure 4.**
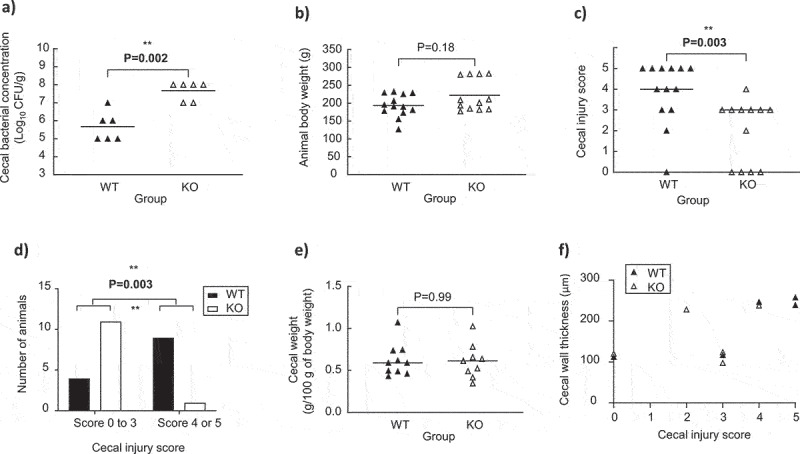
Experimental data of animals’ monoassociated with the wild-type (WT) and knock-out (KO) *C. neonatale* 250.09 strains. (a) Cecal bacterial concentration in quails monoassociated with the WT (n = 6) or KO (n = 6) strains. (b) Body weight of quails monoassociated with the WT (n = 13) or KO (n = 12) strains. (c) Cecal weight of quails monoassociated with the WT (n = 10) or KO (n = 9) strains. (d) Cecal injury score of quails monoassociated with the WT (n = 13) or KO (n = 12) strains. (e) Distribution of the number of quails monoassociated with the WT (n = 13) or KO (n = 12) strains based on the cecal injury score. (f) Correlation between cecal wall thickness and cecal injury score of quails monoassociated with the WT (n = 5) or KO (n = 5) strains. Statistical significance was set at P < .05.

*C. neonatale* 250.09 WT and KO animal experimental data are presented in Figure 4. The KO strain colonized the ceca of quails more than the WT strain (Figure 4a). Body weights at the end of the experiment were the same in the WT and KO groups (Figure 4b) and no quail showed any clinical sign of disease. Similar to the *C. butyricum* assay, the score intensity and the proportion of animals exhibiting at least two NEC-like lesions (scores 4 and 5) were significantly higher in the WT than in the KO group (Figure 4c,d). At the macroscopic level, the lesions observed in the *C. neonatale* 250.09 WT group were hemorrhage foci and wall thickening similar to that for *C. butyricum*. The ceca weight was equivalent in both groups (Figure 4e). Macroscopically, pneumatosis was more extensive in the KO than in the WT groups, with a higher number of large gas pockets in the mesentery in the KO (4/12) than those in the WT (1/13) groups. Examples of ceca are presented in Fig. S2.

**Figure 5.**
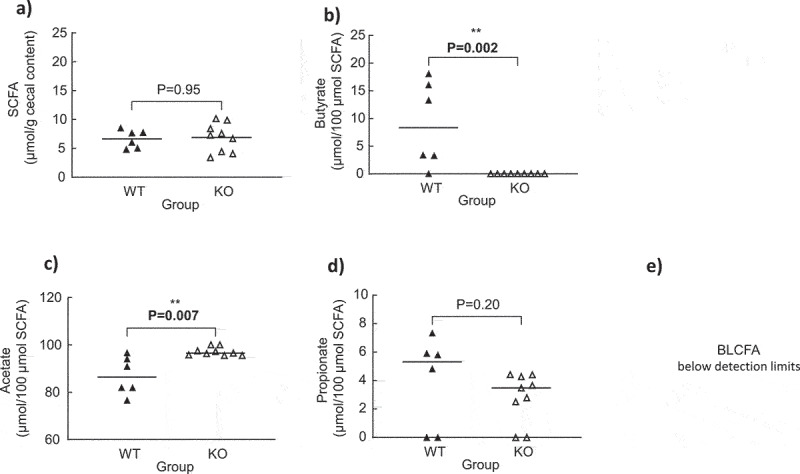
Cecal SCFA, branched long-chain fatty acid (BLCFA), acetate, propionate, and butyrate concentrations in quails monoassociated with the wild type (WT) (n = 6) or knock-out (KO) (n = 9) *C. neonatale* 250.09 stains. Data are individual results with medians. Statistical significance was set at P = .05.

The total cecal SCFA concentrations were similar between the WT and KO groups (Figure 5a). Butyrate was not detected in the cecal content of the quails colonized with the KO strain, whereas butyrate represented 8.3% of total SCFAs in the cecal content of quails colonized with WT strain (Figure 5b). The proportion of acetate was higher in the KO than in the WT groups (Figure 5c). There was no difference in the proportion of propionate between the two groups (Figure 5d), and BLCFA values were below detection limits.

### Cecal histology and electron microscopy

**Figure 6.**
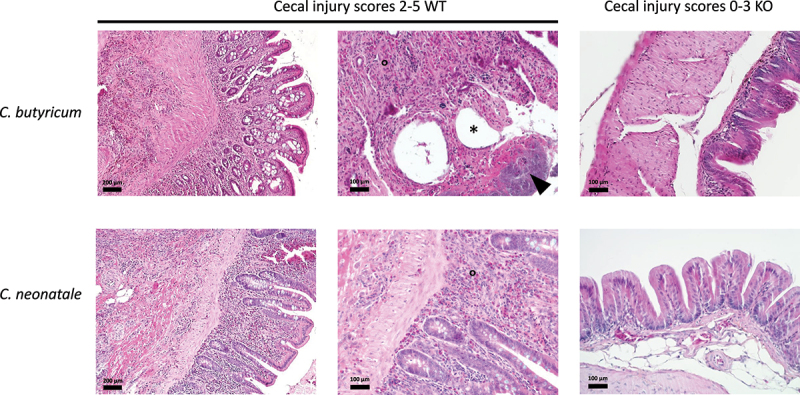
Histopathological presentation of the cecum (hematoxylin–eosin–safranin staining). The ceca infected with *C. butyricum* wild-type (WT) shows severe mucosal thickening secondary to massive mixed inflammatory cell infiltration (°). The focal ulceration (arrowhead) overlaid by bacterial colonies and gas pocket formation (pneumatosis) (*) are highlighted. The ceca infected with the *C. butyricum hbd*-knockout (KO) strain are unremarkable. The WT *C. neonatale* strain also elicited marked mucosal thickening secondary to massive mixed inflammatory cell infiltration of all layers of the cecal wall (°). The ceca infected with *C. neonatale* KO are unremarkable.

Histological analyses were performed in five quails of each *C. butyricum* group. The selected animals corresponded to examples of injury scores of 0–3 and 2–5: injury scores’ medians in these subgroups were close to those of the entire groups. The mean mucosal thickness values of the WT and KO groups were 172 ± 17 µm and 96 ± 7 µm, respectively (P = .007) and were positively associated with injury scores (r = 0.93, P = .003) (figure 2f). Qualitatively, histological analysis in the WT group revealed severe inflammation comprising mononucleated cells and some heterophils that extensively infiltrated all layers of the intestinal wall from the mucosa to serosa (Figure 6). Severe lymphangiectasia was noted. Necrotic foci with cellular debris and ulcerated areas were also observed in the mucosa (Figure 6). In contrast, in the KO group, two quails with a lesion score of 3 displayed multiple foreign body granulomas in the cecal serosa. These granulomas were centered on an optically empty cavity, corresponding to the gas pockets observed macroscopically. These cavities were lined by epithelioid and giant cells; this was not observed in quails in the WT group. No lesion was observed in other animals in the KO group.

Histological analyses of five quails of each *C. neonatale* group (scores 0–5 in the WT group; scores 0–4 in the KO group) indicated similar mean mucosal thicknesses in both groups (195 ± 33 µm and 161 ± 30 µm in the WT and KO groups, respectively; P = .42). As for *C. butyricum*, mucosal thickening strongly and positively correlated with the score lesions (r = 0.75, P = .01) (figure 4f). Histological analyses of the *C. neonatale* WT group revealed severe inflammation throughout the cecal wall, from the mucosa to serosa, comprising degenerating heterophils with some mononucleated inflammatory cell infiltration, similar to that for *C. butyricum*. Necrotic foci with cellular debris and ulcerated areas were also observed (Figure 6). In contrast, inflammation in the ceca in quails in the KO group was mild and mostly restricted to the mucosa with only focal infiltration in the submucosa. Mononuclear cells were primarily present in the mucosa in quails in the KO group, with very few heterophils and the absence of associated ulcerative lesions. Electron microscopy performed on three cecal samples from representative quails inoculated with *C. neonatale* confirmed the presence of bacterial islands around the lesions in the ceca of animals colonized with the WT strains (Fig. S3).

### Characteristics of the wild-type and hbd-knockout strains

**Figure 7.**
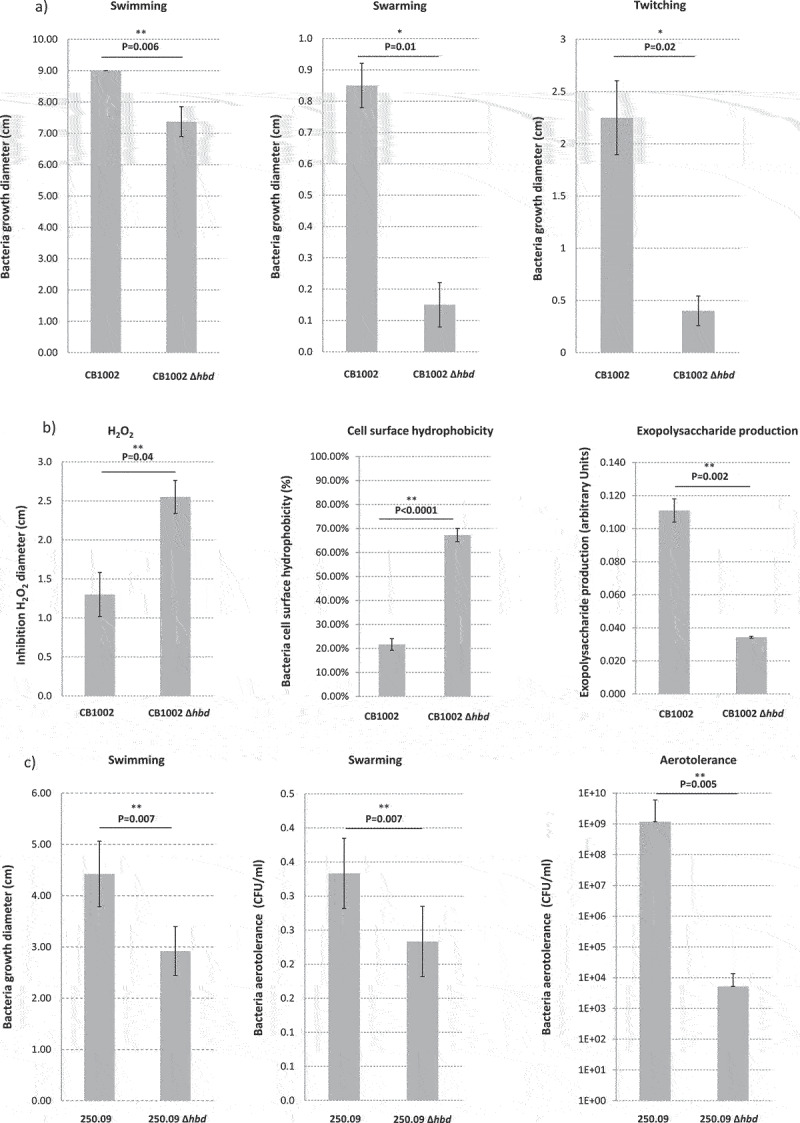
Phenotypic characteristics of the wild type or hbd-knock-out Clostridium butyricum CB1002 (a and b) and Clostridium neonatale 250.09 (c) strains. Data presented are the average of 3 independent experiments.

Only statistically significant differences in terms of phenotypic characteristics of the WT and KO strains are presented in Figure 7. *C. butyricum* CB1002 KO strain showed significantly lower swimming (P = .006), swarming (P = .01), and twitching (P = .02) motility than its WT strain (Figure 7a). *C. butyricum* CB1002 KO strain showed significantly higher resistance to H_2_O_2_ (P = .04) and surface hydrophobicity (P < .0001) and lower exopolysaccharide production (P = .002) than its WT strain (Figure 7b). *C. neonatale* 250.09 KO strain showed significantly lower swimming (P = .007) and swarming (P = .007) motility and lower aero-tolerance than its WT strain (P = .005) (Figure 7c).

## Discussion

In the present study, we chose two different *Clostridium* species previously associated with NEC as a study model and demonstrated that butyrate can influence the development of NEC lesions in a gnotobiotic quail animal model. Our data support the hypothesis that bacterial fermentation of carbohydrates plays a role in NEC development. First, by directed mutagenesis homologous recombination, we successfully inactivated the *hbd* of *C. butyricum* CB1002 and *C. neonatale* 250.09 strains. Both strains were previously isolated from the fecal samples of patients who developed NEC.^20,35,36^ The *hbd* encodes a β-hydroxybutyryl-CoA dehydrogenase that catalyzes the early steps of the biosynthesis of n-butanol from acetyl-CoA, wherein acetoacetyl-CoA is reduced to 3-hydroxybutyryl-CoA that is responsible for the production of end-fermentation metabolites, including butyrate.^37^ At the genetic level, the *hbd* chromosomal disruption was confirmed; at the phenotypic level, *hbd* inactivation resulted in completely blocking the butyrate production without little effect on cell growth. Second, enteropathogenicity of the WT and KO strains was assessed in a gnotobiotic quail model of NEC. The results show that animals fed with a diet supplemented with lactose and colonized with the KO strains developed significantly less macroscopic and microscopic NEC-like lesions. Additionally, SCFA analysis showed the absence of butyrate in the ceca of animals colonized with the KO strains. A previous study by our group suggested that quails fed with a diet deprived of lactose had very low butyric acid production and no digestive lesions.^21,38^ These results highlight the importance of butyrate production by *C. butyricum* and *C. neonatale* from lactose and their enteropathogenic effect and support the metabolic role of the gut bacteria as a component of the events leading to the development of NEC lesions. If total cecal SCFA rates were equivalent in both animal groups, higher yields of some individual SCFA were found in animals colonized with the KO strains such as acetate, propionate, and/or BLCFA. The increased yields of some individual SCFA may reflect the bifurcation of the fermentation pathway of the KO strains when blocking the butyrate production.^37^ Our results are in accordance with some studies that have also shown some adverse effects of butyrate in contrast to its wide range of positive effects on the intestinal mucosa. Indeed, studies reported that rectal administration of butyrate dose dependently increased colonic visceral sensitivity.^39^ In newborn rats, it has been demonstrated that the severity of mucosal injury to butyrate was dose dependent and also depended on the maturation of the intestine.^27,29,30^ Moreover, using cell monolayer model of the intestinal barrier, studies have indicated a paradoxical effect of butyrate with the promotion of the intestinal barrier function at a low concentration and induction of apoptosis and impairment of the intestinal barrier at a high concentration.^27,28^ Indeed, at concentration of 8 mM, butyrate increased the permeability in a Caco-2 cell line.^28^ An *ex vivo* study, using adult rat distal colon mucosa, demonstrated that exposure to butyrate can increase paracellular permeability.^40^ This has also been demonstrated in rats fed a diet-containing fermentable FOS. The rapid bacterial fermentation of FOS led to accumulation of high concentrations of SCFAs that increased intestinal permeability and was associated with increased translocation of *Salmonella*.^41^ Partial purification of *C. butyricum* culture filtrate showed that Caco2 cell cytotoxic factor was related to butyric acid and that cytotoxic titer was dependent upon butyric acid concentration.^32^ Recently, it was suggested that infants with brain injuries had significantly elevated fecal butyrate levels in the first month of life (<28 days) along with *Klebsiella* overgrowth in the gut and a pro-inflammatory immunological tone.^33^

Prematurity of the gastrointestinal functions, such as peristalsis motility, digestive ability, circulatory regulation, barrier function, and immune defense, predisposes the gut to local injury and inappropriate responses to injury. Preterm neonates, in particular those with the lowest gestational age, have insufficient intestinal capacity to digest carbohydrates, such as lactose, owing to the immaturity of their intestinal lactasic equipment.^25^ Therefore, the colonic luminal undigested lactose is available for bacterial fermentation leading to an overproduction of bacterial fermentation-end products. The excessive luminal production of SCFAs, e.g., butyrate, exceeding the physiological capacity of the immature intestinal mucosa of preterm neonates, triggers the inflammatory cascade of NEC.^26,27,29,31^ For instance, butyrate can act positively on IL-10 expression and the epithelial barrier function,^42,43^ but it also aggravates the gastrointestinal tract barrier dysfunction in patients with active inflammation.^44^ In preterm neonates, the potential for injury resulting from insufficient mucus layer protection is heightened by the impaired clearance of luminal contents due to decreased motility and decreased digestion and absorption because of enterocyte immaturity.^45^ This increases the exposure of the vulnerable epithelia to bacteria and toxic luminal contents. In the present study, gnotobiotic quails were used as a suitable experimental model of NEC as it mimics some human preterm neonate characteristics in terms of gastrointestinal physiology. First, the development of NEC-like lesions requires the combination of two major factors present in preterm neonates – lactose in diet and colonization by lactose-fermenting bacteria. Second, NEC develops spontaneously a few weeks after bacterial colonization, similar to preterm neonates who typically develop NEC during the first weeks of life.^2^ Moreover, the induction of NEC-like lesions does not require artificial induction of intestinal hypoxia as opposed to other animal models of NEC.^46^ Third, quails naturally have a very low endogenous lactase activity reflecting preterm neonate immature intestinal lactase activity. Fourth, the ceca of quails are blind-ending ducts favoring physiological stasis of its content, thus mimicking immature intestinal peristalsis of preterm neonates. However, as quails are not mammals, their use as a model for investigating inflammation is limited and was not performed in the present study.

Blocking the butyrate production pathway may have phenotypic alterations on strains and affect pathogenicity. The growth, temperature viability, bile salts, acid, and NaCl tolerance of the KO remained unchanged compared to their WT strains. However, differences were observed concerning flagellar-mediated motility for *C. butyricum* and *C. neonatale* KO strains and pili-mediated motility, cell surface hydrophobicity, exopolysaccharide production, and aero-tolerance for the *C. butyricum* KO. These modifications may have influenced the animal model experimental data. The flagella and pili are considered nonspecific bacterial virulence factors associated with adherence and/or invasion.^47^ However, the cecal colonization of quails with WT or KO strains remained unaffected in the present study. Moreover, *C. butyricum* and *C. neonatale* KO strains had different phenotypic profiles but showed equivalent behavior in terms of enteropathogenicity. We observed that the *C. neonatale* KO strain colonized the ceca of quails more than the WT strain, suggesting that *hbd* mutation may confer some advantage to this species. This needs to be investigated, for instance, through fitness experiments.

Experimental studies on NEC pathogens have been inconclusive because the ability of pathogenic bacteria to cause NEC has yet to be studied systematically and in-depth. If studies have performed clinical and research investigations on NEC, additional key data are needed to understand its physiopathology. This has been emphasized by the Necrotizing Enterocolitis Workgroup of the International Neonatal Consortium, which highlighted the need for significantly reducing the clinical burden of NEC by identifying novel comprehensive hypotheses of the disease.^4^ In the present study, we used strains previously isolated from NEC suffering infants and showed significantly increased clinical scores and NEC lesions in quails and linked the specific inactivation of *hbd* in the same strains leading to a measurable loss in enteropathogenicity. If the presence or the abundance of C. *butyricum* and *C. neonatale* strains and NEC can be debated, our results obtained with this specific animal model provide new mechanistic information on the pathophysiology of NEC and the potential role of bacterial metabolites. Our results provide an alternative mechanism of NEC compared to the model involving the cell wall lipopolysaccharide from *Enterobacteria* that stimulates the innate immature gut immune system through binding to TLR-4 receptors, leading to intestinal inflammation, enterocyte injury, and the onset of NEC.^2,13^ Additionally, the findings support the link between NEC and obligate anaerobes, particularly *Clostridium* species, as an alternative to *Enterobacteria*.

The gap in NEC pathophysiology knowledge has hindered the development of reliable biological markers for efficient diagnosis and prevention. The present study provides new information that may be exploited for developing potential strategies for disease prevention. For instance, implementing a scoring system that considers the gut colonization by *C. butyricum* and/or *C. neonatale* and the evolution of fecal butyrate may be an ideal investigative approach. Our data may also be of interest when speaking of intestinal microbiota modulation in terms of bacteria and metabolites.

## Methods

### Bacterial strains and growth conditions

The strains used in this study are listed in Table S3. Strains were cultured on brain heart infusion (BHI) agar or broth (Oxoid) at 37°C in an anaerobic chamber (Biomerieux, Marcy l’Etoile, France) (v/v: 80% N_2_, 10% H_2_, 10% CO_2_). When necessary, the medium was supplemented with cycloserine (250 mg.mL^−1^), thiamphenicol (15 mg.mL^−1^ to select KO strains or 7.5 mg.mL^−1^ to maintain the plasmid pMTL007-hbd), or erythromycin (5 mg.mL^−1^). *Escherichia coli* TOP10 strain was used for cloning and plasmid propagation. *E. coli* HB101 (RP4) strain was used as the conjugative donor for developing the clostridia KO strains. *E. coli* strains were cultured aerobically at 37°C in Luria–Bertani agar or broth, and when needed, the medium was supplemented with ampicillin (100 mg.mL^−1^) or chloramphenicol (25 mg.mL^−1^).

### C. butyricum *and* C. neonatale hbd *mutagenesis*

The oligonucleotides and plasmids used in this study are listed in Table S1 and S4, respectively. The mobile group II intron system was used as described previously to inactivate the *hbd*.^34,48^ The algorithm available on the TargeTron Design Site (http://www.clostron.com/clostron2.php) was used to identify the intron insertion site within *hbd* (position 414) and design primers to retarget the group II intron in *hbd* (c-hbd-414|415s-IBS, c-hbd-414|415s-EBS1d, and c-hbd-414|415s-EBS2). These primers were used with EBS universal primers and intron template DNA to generate a DNA fragment by overlap PCR as recommended by manufacturers. The PCR product was purified using QIAquick PCR Purification Kit (Qiagen, Courtaboeuf, France) and digested by *Bsr*GI and *Hin*dIII restriction enzymes. Plasmid pMTL007C-E2-*hbd* was developed by ligating (T4 DNA ligase) (Sigma Aldrich Chimie, Saint-Quentin-Fallavier, France) the digested PCR product and pMTL007C-E2 digested with *Bsr*GI/*Hin*dIII and purified with QIAEX II Gel Extraction kit (Qiagen). The ligation mixture was introduced into *E. coli* TOP10 by electroporation. After extracting the plasmid (QIAprep Spin Miniprep kit, Qiagen) and amplifying the insert by FastStart High Fidelity PCR System (Sigma Aldrich Chimie,) with primers pMTLCE2seqF and pMTLseqR, the cloned insert was verified by DNA sequencing using the same primers (Genewiz, Takeley, UK). The plasmid pMTL007C-E2-*hbd* was transformed into the conjugative donor *E. coli* HB101 (RP4) and transferred into the *C. butyricum* 1002 and *C. neonatale* 250.09 strains via conjugation. The transconjugants were selected on BHI agar supplemented with cycloserine and thiamphenicol. Inactivation of *hbd* was verified by screening transconjugants for erythromycin resistance and thiamphenicol sensitivity, and the resultant strains were named CbuCB1002-*hbd*415s::CT and Cne205.09-*hbd*415s::CT. Further, genomic DNA was extracted (InstaGene Matrix Kit) (Bio-Rad, Marnes-la-Coquette, France) and subjected to PCR using primers flanking *hbd* (hbdF and hbdR) to verify the intron insertion into the correct target gene. Moreover, the presence of the *ermB* was confirmed by PCR using primers RAMFCE2 and RAMRCE2, and the orientation of insertion was verified by PCR with a combination of hbdF, hbdR, and EBS Universal primers.

### Phenotypic characterization of strains

Strain motility (swimming, swarming, and twitching), surface hydrophobicity, exopolysaccharide production, aero-tolerance, tolerance to H_2_O_2_ (1% v/v), porcine bile salts (0.3% w/v), acid (pH 4.5), NaCl (5% and 9% w/v), and temperature viability (30°C and 50°C) were assessed as previously described.^49^ Three independent experiments were performed.

### Experiments with quails

Fertilized eggs of a conventional flock of *Coturnix japonica* were obtained from the Poultry Experimental Facility (UE1295 PEAT) of INRAE (Nouzilly, France). They were incubated and aseptically transferred into a sterile hatching isolator two days before the expected hatching day as previously described.^50^ Six days after hatching, the young quails were transferred from the hatching isolator to experimental isolators and inoculated with a freshly prepared culture of a WT *Clostridium* strain (n = 13–15 quails) or of its KO strain (n = 12–15 quails). The WT and KO groups were housed in separate isolators. Inoculation was achieved by orally administering 0.2 mL of the bacterial culture to each quail. Bacterial sterility was checked before inoculation. The bacterial establishment was checked weekly using freshly collected droppings that were observed under an optical microscope and grown in appropriate culture media. Two weeks after bacterial inoculation (quail age: 3 weeks), the starter feed was gradually replaced within 4 days by a grower semisynthetic feed supplemented with 7% lactose (wt/wt) to mimic the proportion of lactose in human milk.^51^ Diets were manufactured by SAFE® diets (Augy, France) and sterilized by γ-irradiation at 45 kGy. The quails received diets and autoclaved drinking water *ad libidum*. Six to eight weeks after inoculation, a sufficient time for the development of NEC-like lesions,^20^ quails aged 7–9 weeks were deeply anesthetized with isoflurane, weighed, and sacrificed by decapitation. The ceca were removed for macroscopic examination and scoring of the lesions (Table S2). The cecal contents were collected and conserved at −80°C until bacterial enumeration and SCFA analysis; however, as the cecal content volume was usually not sufficient to carry out both analyses, some samples were dedicated to bacterial enumeration and the others to SCFA analysis. Empty ceca were weighed to quantify the wall thickening and cecal walls, chosen to represent the variety of macroscopic lesion scores, were fixed in 4% paraformaldehyde, stored at 20°C, and transferred to the APEX histology facility (UMR703 INRAE-Oniris, Nantes, France) for histological analyses. After embedding in paraffin wax, 5-µm-thick transversal ceca sections were stained using the hematoxylin–eosin–safranin staining method. All histological observations were performed blinded by a certified veterinary pathologist. Using low magnification, the mucosal thickness was measured five times per animal cecum on randomly scattered points (intra-assay variation calculated after five independent measurements on the same sample was 7.7%).

Animal experiments were organized as cohorts with simultaneously comparing the WT and KO groups of a given *Clostridium* species, namely *C. butyricum* or *C. neonatale*. Experiments were performed in the Anaxem germ-free animal facility (license number: B78-322-6; INRAE, Micalis Institute, Jouy-en-Josas, France). Procedures conformed to the European guidelines for the care and use of laboratory animals and were approved by the Animal Ethics Committee of the INRAE Research Center, Jouy-en-Josas (approval reference: APAFIS#2540-2015120315579065 v2).

### Biochemical analyses

Gas chromatography was used to measure acetate, propionate, isobutyrate, butyrate, isovalerate, valerate, and phenol levels in cell-free supernatant samples of the KO strains and their respective WT strains, as described previously.^52^ Data were collected from three independent bacterial cultures.

SCFA analysis in the cecal contents was also performed by gas chromatography as described by Lan et al.,^53,54^ with slight modifications. Samples stored at −80°C were thawed at room temperature and extracted with water. Proteins were precipitated with phosphotungstic acid. A 0.3 µL volume of the supernatant was injected onto a gas–liquid chromatogram Agilent 6890 (Agilent Technologies, Courtabeouf, France) equipped with a split-splitless injector, flame-ionization detector, and capillary polyethylene glycol column (15 m × 0.53 mm, 0.5 µm). The carrier gas (H_2_) flow rate was 10 mL/min, and inlet, column, and detector temperatures were 200°C, 100°C, and 240°C, respectively. Between each sample injection, the column was cleaned by increasing the temperature from 100°C to 180°C (20°C/min) followed by holding at 180°C for 2 min before returning to 100°C for the next analysis. We used 2-ethylbutyrate as an internal standard. Samples were analyzed in duplicates. Data were collected and peaks integrated with the OpenLab CDS Chemstation Edition software (Agilent).

### Electron microscopy

Field emission gun scanning electron microscopy (FEG-SEM) was used to observe the cecal walls. Samples were immersed in a fixative solution (2.5% glutaraldehyde in 0.2 M sodium cacodylate buffer, pH 7.4) and stored for 3 h at 20°C and 18 h at 4°C. After removing the fixative, samples were rinsed three times for 15 min in a sodium cacodylate solution (pH 7.4) and were progressively dehydrated in a graded series of ethanol (50% to 100%) before critical-point drying under CO_2_. Samples were mounted on aluminum stubs (50 mm diameter) with carbon adhesive discs (Agar Scientific, Oxford Instruments, Gometz-la-Ville, France) and sputter-coated with platinum (Polaron SC7640, Milexia, Verrières-le-Buisson, France) for 220 s at 10 mA to be visualized by FEG-SEM. They were viewed as secondary electron images (2 kV) with a Hitachi SU5000 instrument (Milexia, Saint-Aubin, France). Sample preparation and analysis were performed in the Microscopy and Imaging Facility for Microbes, Animals, and Foods (MIMA2, INRAE, Jouy-en-Josas, France).

### Statistical analyses

For each *Clostridium* species, the proportions of quails exhibiting NEC-like lesions in the WT and KO groups were compared using Fisher’s exact test. For the other parameters of the animal experiments, the Mann–Whitney U was used. For the phenotypic comparison of the data from the different strains, Welch’s two-sample *t*-test (two-sided) was used. Calculations were performed with GraphPad Prism software (v.7.03, La Jolla, CA, USA). Statistical significance was set at P ≤ .05.

## Supplementary Material

Supplemental Material
