# Supplementary material for: Neonatal necrotizing enterocolitis: Clostridium butyricum and Clostridium neonatale fermentation metabolism and enteropathogenicity
Source: Gut Microbes. 2023 Feb 21;15(1):2172666. doi: 10.1080/19490976.2023.2172666 (PMC9980464; doi:10.1080/19490976.2023.2172666)
Supplement: Supplemental Material [file KGMI_A_2172666_SM4568.docx]

**Supplementary materials**

**Table S1.** Oligonucleotides used in this study.

| **Primers** | **Genetic feature or sequence (3’-5’)** | **Application** |
| --- | --- | --- |
| EBS Universal | CGAAATTAGAAACTTGCGTTCAGTAAAC | *hbd* intron retargeting |
| c-hbd-414\|415s-IBS | AAAAAAGCTTATAATTATCCTTAATAGGCATGCATGTGCGCCCAGATAGGGTG | *hbd* intron retargeting |
| c-hbd-414\|415s-EBS1d | CAGATTGTACAAATGTGGTGATAACAGATAAGTCATGCATTTTAACTTACCTTTCTTTGT | *hbd* intron retargeting |
| c-hbd-414\|415s-EBS2 | TGAACGCAAGTTTCTAATTTCGGTTCCTATCCGATAGAGGAAAGTGTCT | *hbd* intron retargeting |
| pMTLCE2seqF | TAGCCTGTGAAATAAGTAAG | intron sequencing |
| pMTLCE2seqR | CAGATTCTCGGCATCGC | intron sequencing |
| hbdF | TTAGCTGCTGACTGCGATTT | *hbd* mutation verification |
| hbdR | AGGTCCCATTGGGTGGTT | *hbd* mutation verification |
| RAMFCE2F | TATACTTTCTAGAGAATAGGAAC | *hbd* mutation verification |
| RAMFCE2R | CTCTAGAAAGTATAGGAACTTC | *hbd* mutation verification |

**Table S2.** Scoring scale of the animals’ cecal lesions.

| **Score** | **Lesion** | **Severity degree** |
| --- | --- | --- |
| 0 | None | Low or moderate severity |
| 1 | Distension |  |
| 2 | Thickening |  |
| 3 | Pneumatosis (mucosa or/and mesentery) |  |
| 4 | Thickening and pneumatosis  Thickening and haemorrhage foci  Pneumatosis and haemorrhage foci | High severity |
| 5 | Thickening, pneumatosis and haemorrhage or necrosis foci |  |

**Table S3.** Strains used in this study.

| **Strains** | **Genetic feature or sequence (3’-5’)** | **Source, reference, or application** |
| --- | --- | --- |
| ***Escherichia coli*** |  |  |
| TOP10 | F^-^ mcrA Δ(*mrr*-*hsdRMS*-*mcrBC*) ɸ80*lac*ZΔ M15 Δ*lac*X74 *rec*A1 *ara*D139 Δ(ara-leu) 7697 *gal*U *gal*K *rps*L (Str^R^) *end*A1 *nup*G | Invitrogen |
| HB101 (RP4) | *sup*E44 *aa*14 *gal*K2 *lac*Y1 Δ(*gpt*-*proA*) 62 *rps*L20 (Str^R^) *xyl*-5 *mtl*-1 *rec*A13 Δ(*mcr*C-*mrr*) *hsd*S_B_(rB^-^ mB^-^) RP4, Ap^R^ | Laboratory stock |
|  |  |  |
| ***Clostridium butyricum*** |  |  |
| CB1002 |  | Wild-type, isolated from a fatal case of NEC ^50^ |
| CB1002hbd ::erm | CB1002hbd::ermB, Em^R^ | This study |
|  |  |  |
| ***Clostridium neonatale*** |  |  |
| 250.09 |  | Wild-type, isolated from a fatal case of NEC ^36,54^ |
| 250.09hbd::erm | CN250.09hbd::ermB, Em^R^ | This study |

Str^R^, streptomycin-resitant; Ap^R^, ampicillin-resistant; Em^R^, erythromycin-resistant.

**Table S4.** Plasmids used in this study.

| **Plasmids** | **Genetic feature or sequence (3’-5’)** | **Source, reference, or application** |
| --- | --- | --- |
| RP4 | Tra^+^ IncP, Ap^R^, Km^R^, Tc^R^ | ^48^ |
| pMTL007C-E2 | Cm^R^, Tm^R^ | ^48^ |
| pMTL007C-E2-hbd-X | Cm^R^, Tm^R^ | This study |

Ap^R^, ampicillin-resistant; Cm^R^, chloramphenicol-resistant; Tm^R^, thiamphenicol-resistant; Km^R^: kanamycin-resistant; Tc^R^, tetracyclin-resistant.

**Figure S1**. Schematic representation of the clostridia anaerobic carbohydrate fermentation pathway. HBD: β-hydroxybutyryl-CoA déshydrogénase. Red cross: knock-out *hbd*

**HBD**

**GLUCOSE**

**pyruvate**

**Lactate**

**Acetate**

**acetyl-CoA**

**Ethanol**

**acetoacetyl-CoA**

**Acetone**

**3-hydroxybutyryl-CoA**

**butyryl-CoA**

**Butyrate**

**butyryl-P**

LACTOSE

**Figure S2.** Macroscopic examples of the cecum colonized with the *C. butyricum* and *C. neonatale* wild-type (WT) and *hbd*-knockout (KO) strains.


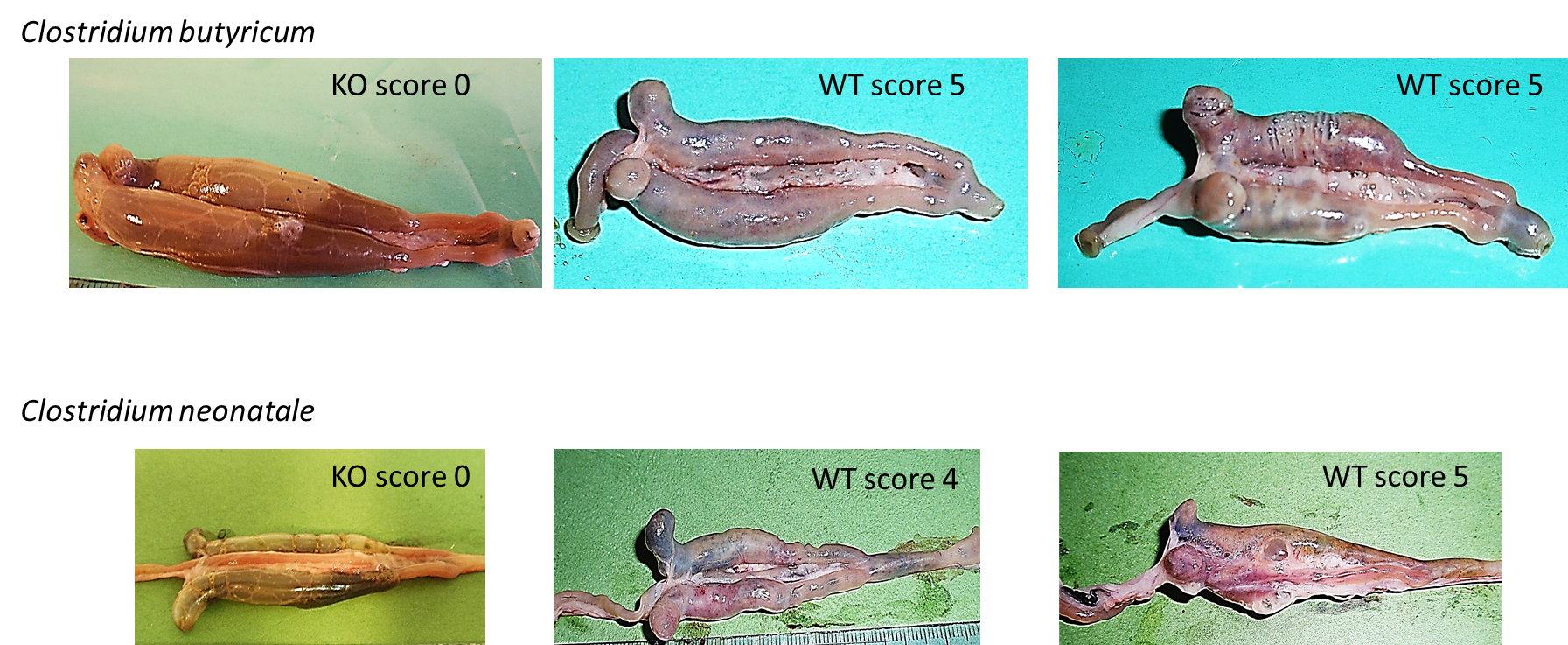


**Figure S3**. Example of field emission gun scanning electron microscopy of quail caeca colonized by the wild-type (A, C, E) or the knock-out (B, D, F) strain of *Clostridium neonatale*  250.09 at different magnifications (A, B x500; C, D x1000; E,F x5000). White arrows show lesions on tissues and black arrows show zones colonized by *C. neonatale*.


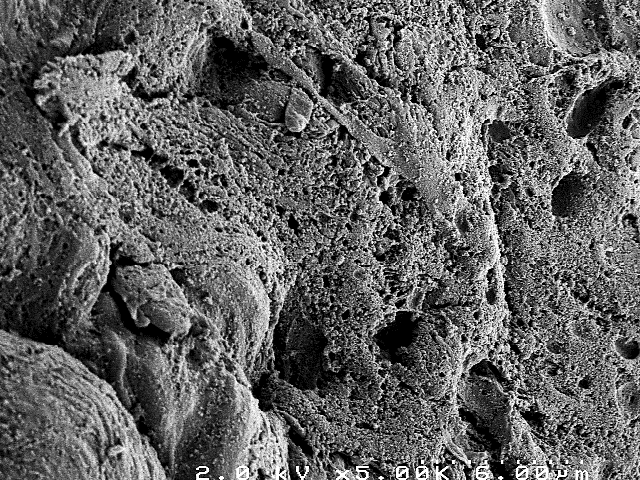


**E**


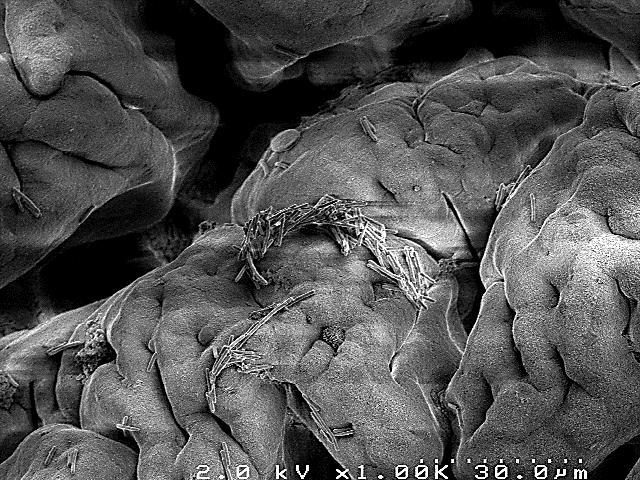


**C**


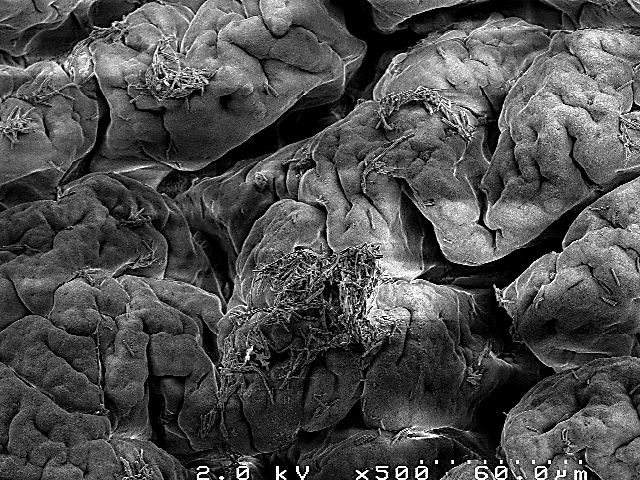


**A**


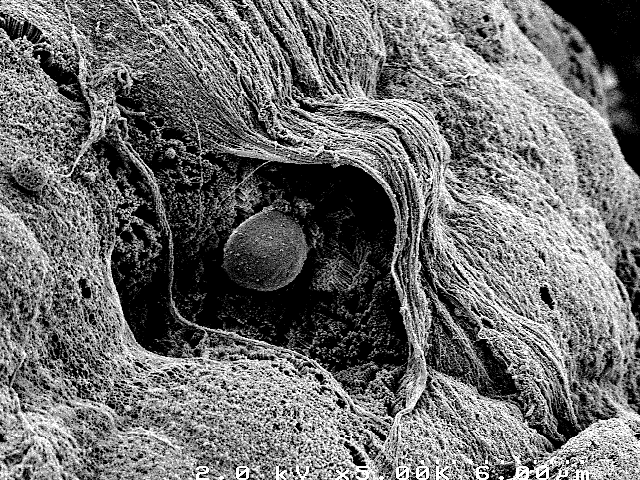


**F**


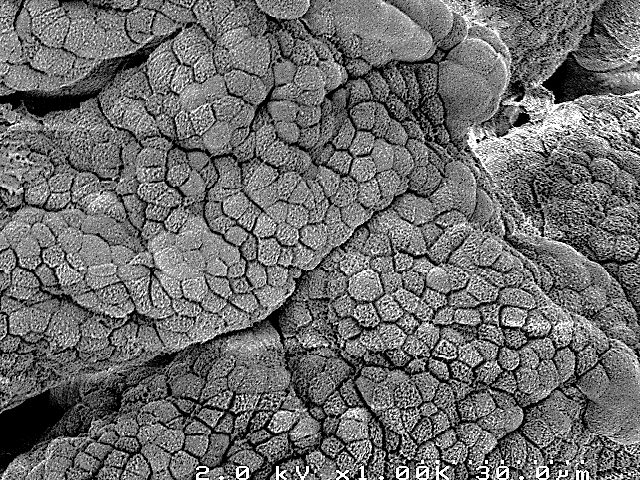


**D**


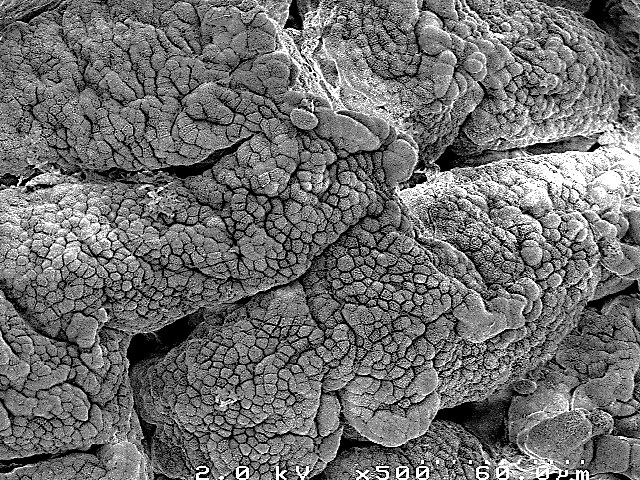


**B**
